# An acoustofluidic scanning nanoscope using enhanced image stacking and processing

**DOI:** 10.1038/s41378-022-00401-2

**Published:** 2022-07-13

**Authors:** Geonsoo Jin, Joseph Rich, Jianping Xia, Albert J. He, Chenglong Zhao, Tony Jun Huang

**Affiliations:** 1 Thomas Lord Department of Mechanical Engineering and Material Science, Duke University, Durham, NC 27708 USA; 2 Department of Biomedical Engineering, Duke University, Durham, NC 27708 USA; 3 Department of Physics, University of Dayton, 300 College Park, Dayton, OH 45469 USA; 4 Department of Electro-Optics and Photonics, University of Dayton, 300 College Park, Dayton, OH 45469 USA

**Keywords:** Micro-optics, Microfluidics

## Abstract

Nanoscale optical resolution with a large field of view is a critical feature for many research and industry areas, such as semiconductor fabrication, biomedical imaging, and nanoscale material identification. Several scanning microscopes have been developed to resolve the inverse relationship between the resolution and field of view; however, those scanning microscopes still rely upon fluorescence labeling and complex optical systems. To overcome these limitations, we developed a dual-camera acoustofluidic nanoscope with a seamless image merging algorithm (alpha-blending process). This design allows us to precisely image both the sample and the microspheres simultaneously and accurately track the particle path and location. Therefore, the number of images required to capture the entire field of view (200 × 200 μm) by using our acoustofluidic scanning nanoscope is reduced by 55-fold compared with previous designs. Moreover, the image quality is also greatly improved by applying an alpha-blending imaging technique, which is critical for accurately depicting and identifying nanoscale objects or processes. This dual-camera acoustofluidic nanoscope paves the way for enhanced nanoimaging with high resolution and a large field of view.

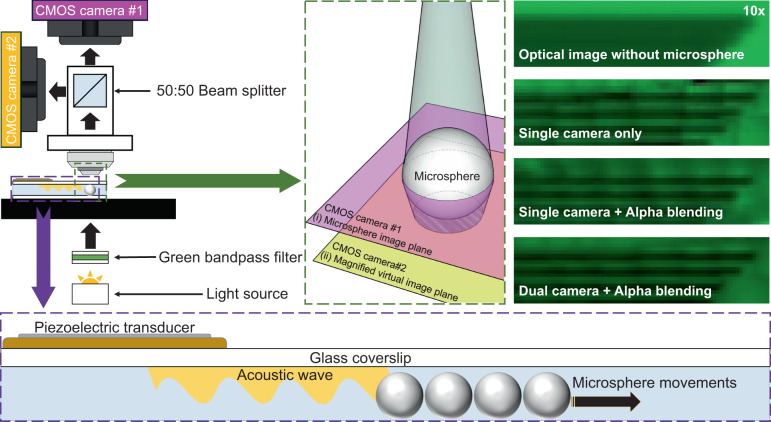

## Introduction

Optical microscopy has become an indispensable tool in the fields of biology, medicine, chemistry, and physics due to its rapid and noninvasive imaging capabilities^1,2^. However, the diffraction limit of light from a conventional microscope places an upper limit on its maximum resolution^3,4^. In addition, the field of view of a conventional microscope is typically inversely proportional to its resolution. In other words, higher resolution imaging is obtained at a reduced field of view, and larger field-of-view imaging is achieved at a lower optical resolution. Various imaging technologies have been developed to increase the optical resolution beyond the diffraction limit, such as stochastic optical reconstruction microscopy^5–7^, photoactivated localization microscopy^8–10^, stimulated emission depletion imaging^11–13^, structured illumination microscopy (SIM)^14–16^, and nanospeckle illumination microscopy (NanoSIM)^17,18^. However, the inverse relationship between optical resolution and the field of view still exists in these advanced microscope systems, and these systems are limited by complex optical setups or by relying on fluorescent labeling.

To break the diffraction limit of light without using fluorescent labeling, optical imaging with the help of a dielectric microsphere has become a viable solution due to the so-called photonic nanojet effect from the microsphere. The photonic nanojet effect is affected by both the microsphere diameter and the refractive index of the microsphere^19–23^. Essentially, microspheres of a certain size and refractive index can enhance optical imaging to overcome the light diffraction limit. Therefore, a microsphere can be directly placed over the target specimen and act as a superresolution lens to amplify and increase the resolution of an optical image from a conventional microscope^24–32^. An optical resolution as small as 50 nm has been demonstrated with this method^33^. The easy-to-implement nature of this method makes it an attractive and affordable way to boost the resolution of a conventional microscope^34^. However, the superior resolution of this method is achieved at a significantly reduced field of view, which is essentially reduced to the size of the microsphere. Although the field of view can be potentially increased by attaching a microsphere to an atomic force microscope (AFM) cantilever to scan the sample surface^35^, the mechanical scanning of an AFM cantilever renders the imaging process slow and sensitive to outside vibrations.

Recently, we demonstrated a single-camera acoustofluidic scanning nanoscope to overcome the aforementioned limitations^36^. In this method, multiple microspheres are simultaneously driven by acoustic forces to scan a target sample^37–56^. The superresolution image from each microsphere can be merged together to generate a large field of view. The accurate determination of the microsphere position plays an important role in the imaging process, where a circle-finding algorithm is applied to locate the microspheres. However, the accuracy of the circle-finding algorithm depends on the image quality of the microspheres, which is blurred when the imaging system focuses on the sample. The blurred image of the microspheres therefore introduces errors in determining the particle position and leads to a deteriorated image quality and reduced field of view.

To overcome this limitation, we introduce a dual-camera acoustofluidic scanning nanoscope with a seamless image merging algorithm (alpha-blending process) to significantly improve the image quality. Compared to a single-camera acoustofluidic scanning nanoscope, the dual-camera configuration with an automated alpha-blending image processing algorithm significantly improves the imaging process as follows: (1) the dual-camera configuration reduces the required images to form a large field of view by more than 55-fold compared to the single-camera configuration, which equates to an ~60-fold reduced image processing time; (2) the dual-camera configuration significantly improves the microsphere tracking accuracy, as both the sample and the microspheres can be precisely imaged simultaneously on the dual cameras. In contrast, the single-camera configuration can only precisely image the sample. (3) The dual-camera configuration also allows us to improve the image quality of the sample by applying an automated alpha-blending image processing algorithm.

## Results and discussion

### Configuration of the dual-camera acoustofluidic nanoscope

**Fig. 1:**
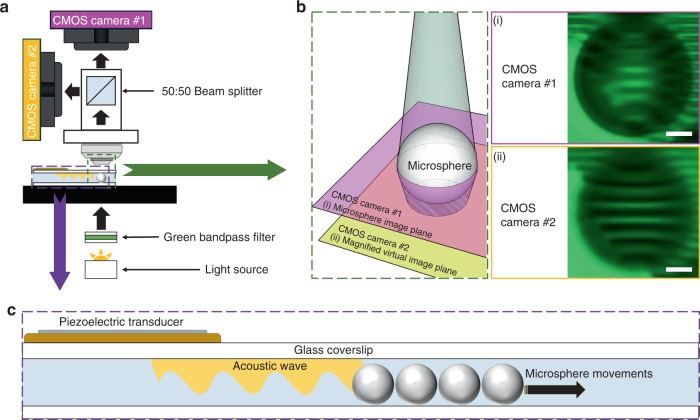
Schematic figure of the dual-camera acoustofludic nanoscope. **a** A 50:50 beam splitter delivered two image planes with two different focal points. A transmitted white light source with a green bandpass filter delivered illumination with minimized chromatic aberration. **b** Schematic and experimental result of the dual-camera imaging planes: (i) Camera #1 focused at the center height of microspheres, as shown by the pink focal point, (ii) Camera #2 focused on the virtual image plane, as shown by the yellow focal point. Each scale bar is 5 µm. **c** Schematic figure of the acoustofluidic nanoscope device. A circular-shape piezoelectric transducer is bonded onto a glass cover slip. Induced acoustic energy pushed microspheres to scan the sample surface.

Figure 1a shows the configuration of the dual-camera acoustofluidic nanoscope system. The system is built on top of an upright Nikon microscope. A 20× objective lens (NA = 0.5) is used for imaging because it can acquire the target sample resolution while maintaining the largest field of view. Two cameras (CMOS cameras #1 and #2) are mounted at two separate imaging ports, as shown in Fig. 1a. A 50:50 beam splitter is inserted into the system to route the light into the two cameras. The positions of the two cameras are adjusted so that camera #1 images the microsphere while camera #2 images the sample. Therefore, the dual-camera configuration allows us to clearly image both the microspheres (Panel (i) in Fig. 1b) and the sample (panel (ii) in Fig. 1b) simultaneously. Here, a sample consisting of 125 gratings with a line-to-line distance of 800 nm is used for the experiment. Microspheres with a diameter of 20 µm were placed on the sample and imaged through the microspheres with a 20× objective lens. Panel (i) of Fig. 1b shows the image plane of camera #1 focused on the microsphere. Focusing on the microspheres clearly outlines the microsphere boundaries and enables accurate tracking of the microspheres with the circle-finding algorithm. Panel (ii) of Fig. 1b shows the image plane of camera #2 focused on the magnified virtual image of the sample. Focusing on the sample clearly images the line structure of the sample. However, focusing on the sample also blurs the boundary of the microspheres. This is what typically occurs in a single-camera imaging configuration (with only camera #2), which makes it difficult to accurately track the microsphere. The dual-camera configuration allows us to find and track the microspheres more precisely; therefore, it can more efficiently preserve both the moving location of the particles and the image quality of the magnified area of interest than the single-camera configuration. In contrast, in the single-camera configuration, the number of microspheres that can be tracked on the sample surface is restricted due to the blurred image of the microspheres. Figure 1c shows a schematic configuration of an acoustofluidic device manipulating the movements of the microspheres. The acoustofluidic device is fabricated by a circular shaped piezoelectric transducer with a glass cover slip. A diluted solution of microspheres with deionized water is placed onto the sample surface and covered by the fabricated device, as shown in Fig. 1c. A circular-shape piezoelectric transducer bonded onto a cover glass was used. Induced acoustic waves from the acoustofluidic device generate acoustic pressure inside the water channel so that microspheres are pushed to scan the sample surface.

### Acoustofluidic scanning of microspheres

**Fig. 2:**
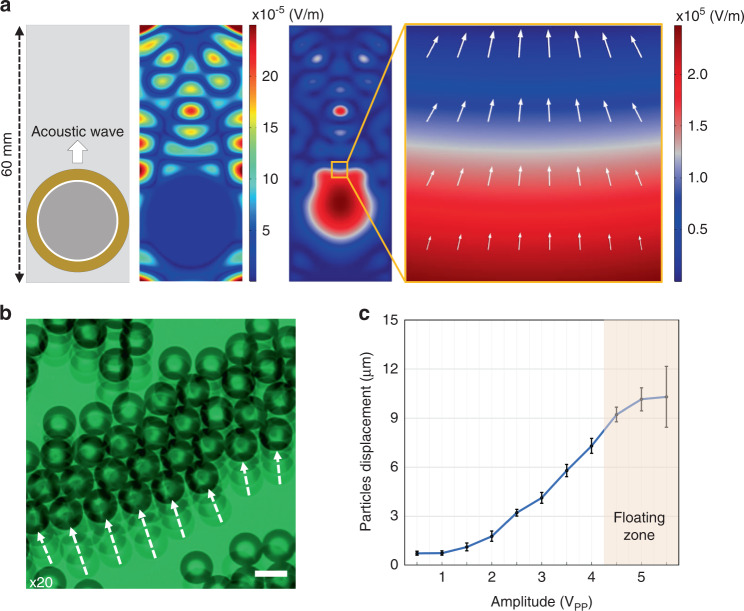
Acoustofluidic particle manipulation for 2D scanning. **a** Left: Schematic of the acoustofluidic device consisting of a circular shaped piezoelectric transducer with a glass cover slip. **a** Middle: Simulation result of the acoustic pressure on the bottom surface of the acoustofluidic device. **a** Right: Simulation result of the acoustic pressure within the water medium below the glass. The small orange box indicates the formation of an acoustic streaming point to uniformly push the microspheres. **b** Microsphere particle movement as a function of input acoustic amplitude applied with a 2.1 kHz wave at a 0.2 s burst interval. The scale bar is 20 µm. **c** Particle displacement distance with a given applied amplitude. At an amplitude of 4.5 V_PP_ and higher (orange area), microspheres were observed to float in suspension. *N* = 100 tracked particles, and the error bars represent the standard deviation.

To obtain a large field of view of the sample, we utilized an acoustofluidic scanning method to manipulate the microspheres so that each area of the sample could be imaged by the microspheres. A circular shaped piezoelectric transducer was bonded onto a 150 μm-thick glass cover slip to actuate acoustic waves, as shown in the left panel of Fig. 2a. Device fabrication is further described in the “Experimental section”. We first optimized the location of the effective scanning area by mathematically simulating the device with the corresponding environment. The simulated acoustic energy distribution on the surface of a glass at a frequency of 2.1 kHz is shown in the middle panel of Fig. 2a. The simulated acoustic pressure in the water above the glass is shown in the right panel of Fig. 2a. From this simulation result, we selected the area of interest for particle manipulation, as shown in the small orange box in the right panel of Fig. 2a. The imaging area of the orange box was selected to be near the transducer without overlapping it. As per the simulation result in the right panel of Fig. 2a, microspheres could be manipulated over a large area (3620 × 3620 µm) across the high and low acoustic pressure areas in the simulation results. Within the enlarged orange box, the acoustic pressure created a near uniform travel direction (white arrows) for the particles to translate when the standing acoustic wave was applied. Figure 2b shows the stacked images of the movement of the particles when we applied a sine wave with a burst mode of 0.2 s intervals to the piezoelectric transducer (Video S1 in the Supporting Information). The input frequency was set to the transducer resonance frequency of 2.1 kHz, and the amplitude was optimized for the most efficient scanning performance. We swept the amplitude from 0.5 to 5.5 peak-to-peak voltage (V_PP_), and the particle movements were measured by the TrackMate function in ImageJ software and are depicted in the graph of Fig. 2c. We selected 100 moving particles in each amplitude experiment and averaged the total moving distance. The error bars in the graph indicate the standard deviation of particle movement. From this experiment, we found that at an amplitude higher than 4.0 V_PP_, the microspheres started to float, thereby preventing an efficient scanning process (Fig. S1 in the Supporting Information). Thus, we selected an amplitude of 4 V_PP_ to drive the microspheres a distance of 7 ± 0.45 µm for the 0.2 s interval without floating.

### Enhanced image tracking using dual-camera imaging

The microspheres allow us to obtain a well-resolved and magnified image of the sample that would not be possible without the microspheres, as shown in Fig. 1b. Together with the acoustofluidic scanning method discussed above, a large field-of-view image can be obtained by scanning the microspheres across the area of interest of the sample. The dual-camera configuration shown in Fig. 1a allows us to accurately track the position of each microsphere and sample image. To perform the scanning process, we collected images of both the sample and the microspheres. Camera #2 records clear images of the sample through the microspheres, while camera #1 records clear images of corresponding microspheres appearing in the field of view of Camera #1, as shown in Fig. 1b. A circle-finding algorithm is applied to the images of the microspheres recorded on camera #1 to determine the central position of each microsphere (x- and y-coordinate pixel values). The radius of each microsphere can also be obtained in this process. The central position and the radius of each microsphere on the image of camera #1 can be replicated to the corresponding sample images in camera #2 by taking into account a magnification factor. The magnification factor is determined to be 0.984 by calibrating the two cameras from the known pitch of the sample.

### Seamless image merging with alpha-blending

**Fig. 3:**
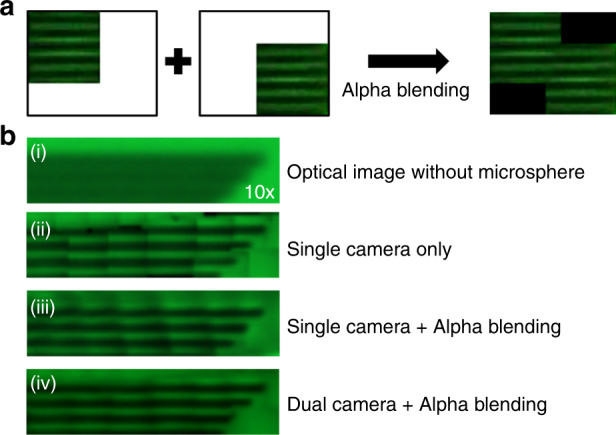
Seamless image merging algorithm. **a** Alpha-blending process in two different positions of the sample. After the alpha-blending process, the overlapped edge boundary is much smoother. **b** Image quality comparison utilizing different imaging methods with alpha-blending in an 800 nm grating line’s structure sample. (i) Optical image without microspheres with a 10× objective lens could not resolve the sample. (ii) Microsphere imaging with a single-camera acoustofluidic nanoscope without alpha blending. (iii) Microsphere imaging with a single-camera acoustofluidic nanoscope with alpha blending. (iv) Microsphere imaging with a dual-camera acoustofluidic nanoscope with alpha blending showed a clear scanned image of the sample.

The images from each microsphere have to merge together to form a large field-of-view image. The image from each microsphere is cropped and overlayed together according to their central positions. An alpha-blending technique, which is frequently used within the image processing field^57–59^, is applied to smooth the boundary between two adjacent images. We integrated this method into our dual-camera acoustofluidic system by applying the alpha-blending technique to a scanned area of the sample from a microsphere, as shown in Fig. 3a. Figure 3b shows the comparison of the final images with different types of imaging and merging methods. Panel (i) of Fig. 3b shows that the grating sample could not be resolved when we used a 10x objective lens without microspheres. A 10× objective lens was utilized to demonstrate the change more effectively in enhanced resolution with the microspheres. For example, in panels (ii) to (iv), we can resolve the grating lines when applying the microsphere imaging method. In panel (ii) of Fig. 3b, a single-camera imaging method was applied without the alpha-blending method and shows a merged scanned image that is not accurately aligned. The reason for this misalignment is that the circle-finding algorithm in the single-camera image is slightly malfunctioned since the circles have unclear boundary edges. In Panel (iii) of Fig. 3b, the alpha-blending applied single-camera image method shows a much smoother image but still shows a slightly misaligned result. In contrast, the dual-camera acoustofluidic nanoscope with an applied alpha-blending technique delivered a finely aligned, clear scanned image in Panel (iv) of Fig. 3b. Although there are still some imperfections in the image quality and a higher image frame rate could be applied to average a higher number of images to provide a clearer image, by applying the alpha-blending technique, the target sample can be clearly imaged with the minimal number of frames.

### Enhanced imaging with a large field of view

**Fig. 4:**
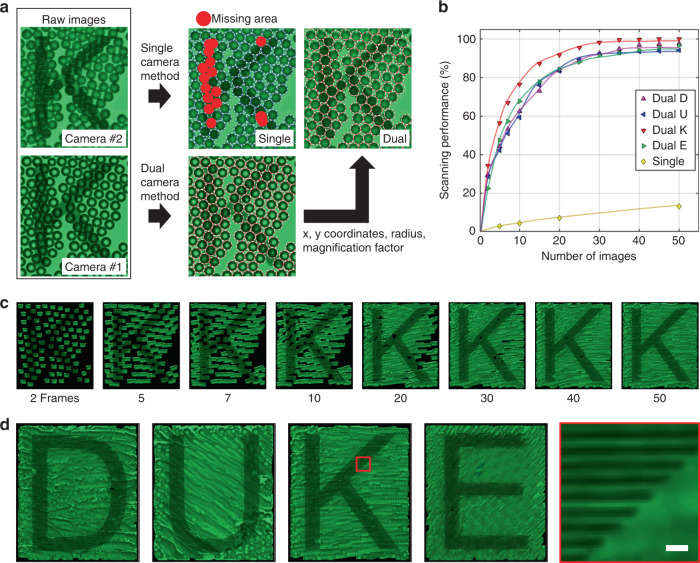
2D scanned images by single-camera and dual-camera acoustofluidic scanning nanoscopes. **a** Image tracking result comparison between single-camera imaging and the dual-camera imaging methods. Collected information (x-, y-coordinates, radius, magnification factor) from the image of camera #1 was applied to the image of camera #2. The red missing area was detected when the single-camera imaging method was applied. **b** The scanning performance comparison between the single-camera and dual-camera imaging methods. Scanning performance was evaluated for the letter for each number of image frames for both imaging methods. **c** Scanned letter “K” images composed of different numbers of image frames from 2 to 50. The letter comprised 800 nm chrome gratings pitched at the same size. **d** Scanned 2D images of the letters “DUKE” by using 20 µm polystyrene microspheres with a 20× microscope objective. The red box indicates the enlarged scanning area in the letter “K”. The scale bar is 2 µm.

A clear image with a large field of view can be readily obtained by combining precise image tracking *via* the dual-camera configuration and smooth image merging *via* alpha blending. We also found that this method saves data collection time and processing time by 55 times compared to that with a single-camera configuration due to the enhanced pixel contrast of the microsphere edge for rapid identification of the microsphere location, thereby increasing the number of magnified images attained from each individual microsphere. To verify the scanning performance, we prepared a chrome–glass mask with the letters ‘DUKE’ that consists of 800 nm pitch and width grating lines. As depicted in Fig. 4a, the microsphere location is better preserved with the dual-camera configuration. The dual-camera acoustofluidic nanoscope allows us to precisely track the position of all the images in the field of view. In contrast, single-camera acoustofluidic imaging can only track 87% of images of the sample. We evaluated the scanning performance of the dual-camera and single-camera imaging configurations, as shown in Fig. 4b. Here, the scanning performance is defined as the percentage of the area scanned of the field of view by the microsphere images. The result of the single-camera imaging method in Fig. 4b was calculated from the result of the previous acoustofluidic nanoscope, which utilized a single-camera-based scanning method^36^. Less than 20% of the field of view was scanned from 50 images of the sample in the previous single-camera imaging method. On the other hand, 99% of the field of view was scanned from 50 images of the sample in the dual-camera configuration. Figure 4c shows how the scanned letter “K” was generated with the increase in image frames. It took 50 image frames and ~10 s to scan 99% of the 200 × 200 μm area for the letter “K”. The 50 image frames were processed by a Python script within 60 s to form the final scanned image. Figure 4d shows the final image for the four letters “DUKE” processed with this method. Each processed image started with approximately 150 microspheres. The dual-camera configuration is ~55 times more efficient than the single-camera configuration. The efficiency is calculated by the ratio between the required number of images to scan 99% of the field of view in the single-camera (3500 images) and dual-camera methods (63.75 on average images in 4 letters). Table 1 in the Supplementary Information compares the resolution and field of view of the enhanced acoustofluidic scanning nanoscope system and other superresolution imaging systems.

## Conclusion

In this work, we demonstrated the enhanced large-field-of-view imaging of an acoustofluidic nanoscope with a dual-camera configuration and a seamless image merging algorithm (alpha-blending process), which can enhance the resolution and enlarge the field of view of a conventional optical microscope. Acoustically driven microspheres on the target sample surface could provide not only superresolution images by the photonic nanojet effect but also 2D scanned images by compiling the traces of the microsphere movements. The enhanced dual-camera imaging acoustofluidic nanoscope achieved high resolution with large-field-of-view imaging with 55 times fewer image frames. Moreover, the dual-camera configuration delivered precise microsphere tracking, which showed clear scanned image boundaries by the alpha-blending technique. This increase in scanning performance and data imaging time could extend the acoustofluidic nanoscope applications to rapidly image and identify dynamic biological systems.

## Experimental section

### Optical characterization

As shown in Fig. 1a, we installed two CMOS cameras (DFK 33UX264, Imagingsource, USA) on an upright microscope (Eclipse LV100, Nikon, Japan) and imaged them with a 20× objective lens (NA: 0.5, Nikon, Japan). Those cameras captured 5 mega-pixel images at 38 frames per second. At this speed, we could perform particle manipulation with 0.2 s acoustic burst intervals. To capture two images at the same time, we placed a 50:50 beam splitter (CCM1-BS013, Thorlabs, USA) at the point of intersection between the two cameras. Since microspheres are not optimized for chromatic aberration and to avoid chromatic aberration, we utilized a green bandpass filter (FB530-10, Thorlabs, USA) and attached a white light source to illuminate the sample.

### Acoustofluidic device fabrication

A circular shaped piezoelectric transducer (AB2720B-LW100-R, PUI Audio, Inc., USA) was bonded onto a 150 µm thick cover glass (24 × 60 mm No. 0-3223, Erie Scientific LLC., USA) with epoxy bonding (PermaPoxyTM 5 min General Purpose, Permatex, USA).

### Microsphere preparation and experimental setup

To perform superresolution imaging with the microspheres, we chose 20 µm polystyrene microspheres (refractive index: 1.6, Sigma-Aldrich, USA). The microspheres were diluted with deionized water before being placed on the sample surface. The microsphere concentration was adjusted to have the maximum number of microspheres within a single monolayer of the field of view to maximize the number of target sample images per frame and reduce the errors introduced by overlapping microspheres. To maintain a consistent water channel height between the device and sample, a square cover glass (#1.5, 10 × 10 mm, Ted Pella, USA) was placed at both ends of the device. The MATLAB (version: R2020b) script was designed and executed to control the function generator (FY6600, FeelTech, China) and CMOS cameras simultaneously to collect the image data. An acoustic burst mode with 0.2 s intervals was applied, and the image acquisition for the two CMOS cameras was executed between the 0.2 s intervals.

### Acoustic streaming simulation

To understand the acoustic streaming within the device, a model of an acoustic device was designed in COMSOL Multiphysics^®^. The model includes the piezoelectric transducer, cover glass, and water under the cover glass. A time domain study was used to visualize the transducer excitation. A 2.1 kHz and 4 V_PP_ signal were applied to the transducer for the electrostatics module. A low reflection boundary water layer with open channel conditions was applied to the cover glass layer. We observed the vibration profile and acoustic streaming to locate the proper microsphere manipulation area, which was ~300 μm away from the transducer.

### Imaging sample preparation

To experimentally demonstrate the scanning performance of the system, we fabricated a chrome patterning sample with the words “DUKE” on a glass mask (Micro Lithography Services Ltd, UK). Each letter is 200 × 200 µm in size and is composed of vertical line gratings 800 nm in width and pitch.

### Image processing method

To generate a final scanned image, the collected images were processed in the following steps. First, a circle-finding algorithm was executed in the image of Camera #1, as seen in Panel (i) of Fig. 1b, in which information on the microsphere coordinates and radius were stored. The magnification factor was calculated by the length of the sample grating line pitch ratio between Camera #1 and Camera #2. Then, the calculated magnification factor (0.984) was multiplied into the coordinates and radius information and applied to the images from Camera #2, as shown in Panel (ii) of Fig. 1b. Next, the microsphere magnified circle images were cropped from the images of Camera #2. Finally, the cropped images were pasted onto the final image with an alpha-blending technique to smooth the boundaries between images. Then, each image was recursively processed in the same manner. The final scanned image was generated from the repetitive image processing algorithm.

## Supplementary information

Supplemental Material
