# Supplementary material for: An acoustofluidic scanning nanoscope using enhanced image stacking and processing
Source: Microsyst Nanoeng. 2022 Jul 13;8:81. doi: 10.1038/s41378-022-00401-2 (PMC9279327; doi:10.1038/s41378-022-00401-2)
Supplement: Supplementary file 1 — Supplemental Material [file 41378_2022_401_MOESM1_ESM.docx]

Supporting Information

**Acoustofluidic scanning nanoscope *via* enhanced image stacking and processing**

*Geonsoo Jin,^1^ Joseph Rich,^2^ Jianping Xia,^1^ Albert He, ^1^ Chenglong Zhao^3^,^4^* and Tony Jun Huang^1*^*

^1^ Thomas Lord Department of Mechanical Engineering and Material Science, Duke University, Durham, North Carolina 27708, United States

^2^ Department of Biomedical Engineering, Duke University, Durham, NC 27708, United States

^3^ Department of Physics, University of Dayton, 300 College Park, Dayton, Ohio 45469, United States

^4^ Department of Electro-Optics and Photonics, University of Dayton, 300 College Park, Dayton, Ohio 45469, United States

*Email: czhao1@udayton.edu; tony.huang@duke.edu

**
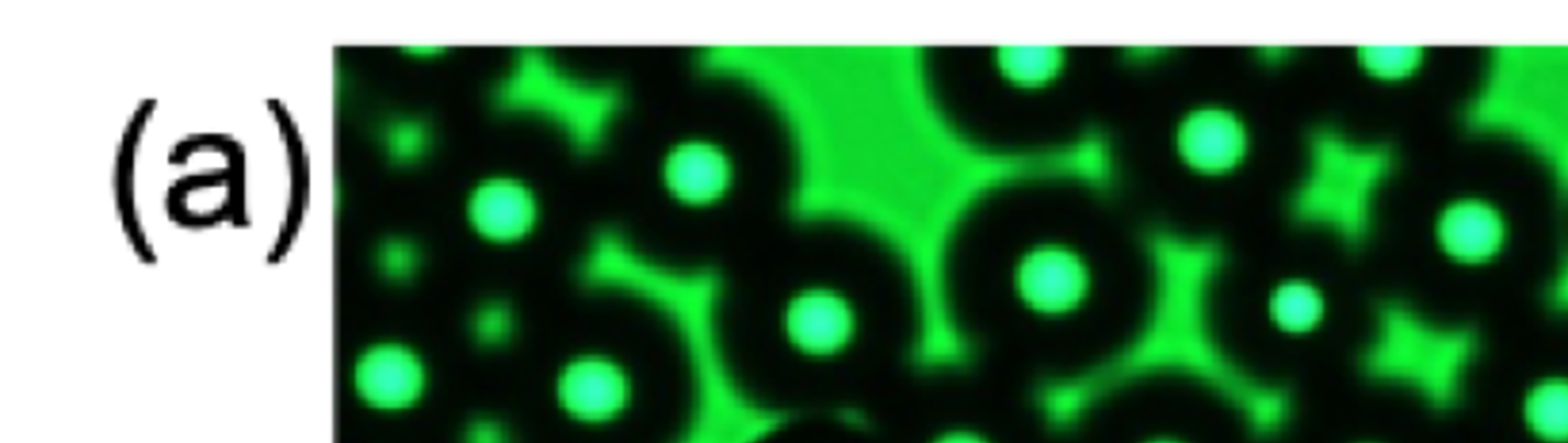
**

**Figure S1.** Microspheres floating when a voltage higher than 4 V_PP_ is applied from a function generator. (a) 4.5 V_PP_, (b) 5.0 V_PP_ and (c) 5.5 V_PP_. Scale bar is 20 µm.

**Table 1**: Comparison of the resolution and field of view of the enhanced acoustofluidic scanning nanoscope and other super-resolution imaging systems. 150 nm of lateral resolution is referred to our previous experimental data which involves detection of nanostructure on a Blu-ray disc.^1^

**Video SV1.** Microparticles movement by the enhanced acoustofluidic scanning nanoscope. We applied 4 V_PP_, 2.1 kHz, and 0.2 sec interval burst to the acoustofluidic device.

**REFERENCES**

1 Jin, G. *et al.* Acoustofluidic Scanning Nanoscope with High Resolution and Large Field of View. *ACS Nano* (2020).

2 Dixon, A., Damaskinos, S. & Atkinson, M. A scanning confocal microscope for transmission and reflection imaging. *Nature* **351**, 551 (1991).

3 Betzig, E. & Trautman, J. K. Near-field optics: microscopy, spectroscopy, and surface modification beyond the diffraction limit. *Science* **257**, 189-195 (1992).

4 Shroff, H., Galbraith, C. G., Galbraith, J. A. & Betzig, E. Live-cell photoactivated localization microscopy of nanoscale adhesion dynamics. *Nature methods* **5**, 417-423 (2008).

5 Manley, S. *et al.* High-density mapping of single-molecule trajectories with photoactivated localization microscopy. *Nature methods* **5**, 155-157 (2008).

6 Annibale, P., Vanni, S., Scarselli, M., Rothlisberger, U. & Radenovic, A. Identification of clustering artifacts in photoactivated localization microscopy. *Nature methods* **8**, 527-528 (2011).

7 Hell, S. W. & Wichmann, J. Breaking the diffraction resolution limit by stimulated emission: stimulated-emission-depletion fluorescence microscopy. *Optics letters* **19**, 780-782 (1994).

8 Hein, B., Willig, K. I. & Hell, S. W. Stimulated emission depletion (STED) nanoscopy of a fluorescent protein-labeled organelle inside a living cell. *Proceedings of the National Academy of Sciences* **105**, 14271-14276 (2008).

9 Farahani, J. N., Schibler, M. J. & Bentolila, L. A. Stimulated emission depletion (STED) microscopy: from theory to practice. *Microscopy: science, technology, applications and education* **2**, 1539-1547 (2010).

10 Olarte, O. E., Andilla, J., Gualda, E. J. & Loza-Alvarez, P. Light-sheet microscopy: a tutorial. *Advances in Optics and Photonics* **10**, 111-179 (2018).
